# Face-selective responses in combined EEG/MEG recordings with fast periodic visual stimulation (FPVS)

**DOI:** 10.1016/j.neuroimage.2021.118460

**Published:** 2021-11-15

**Authors:** O. Hauk, G.E. Rice, A. Volfart, F. Magnabosco, M.A. Lambon Ralph, B. Rossion

**Affiliations:** a MRC Cognition and Brain Sciences Unit, University of Cambridge, 15 Chaucer Road, Cambridge CB2 7EF, UK; b Université de Lorraine, CNRS, CRAN UMR 7039, Nancy F-54000, France; c Research Institute for Psychological Science, University of Louvain, Louvain-la-Neuve, Belgium; d Université de Lorraine, CHRU-Nancy, Service de Neurologie, Nancy F-54000, France

**Keywords:** Face recognition, Face categorization, Frequency tagging, Magnetoencephalography, Electroencephalography, Source estimation

## Abstract

Fast periodic visual stimulation (FPVS) allows the recording of objective brain responses of human face categorization (i.e., generalizable face-selective responses) with high signal-to-noise ratio. This approach has been successfully employed in a number of scalp electroencephalography (EEG) studies but has not been used with magnetoencephalography (MEG) yet, let alone with combined MEG/EEG recordings and distributed source estimation. Here, we presented various natural images of faces periodically (1.2 Hz) among natural images of objects (base frequency 6 Hz) whilst recording simultaneous EEG and MEG in 15 participants. Both measurement modalities showed face-selective responses at 1.2 Hz and harmonics across participants, with high and comparable signal-to-noise ratio (SNR) in about 3 min of stimulation. The correlation of face categorization responses between EEG and two MEG sensor types was lower than between the two MEG sensor types, indicating that the two sensor modalities provide independent information about the sources of face-selective responses. Face-selective EEG responses were right-lateralized as reported previously, and were numerically but non-significantly right-lateralized in MEG data. Distributed source estimation based on combined EEG/MEG signals confirmed a more bilateral face-selective response in visual brain regions located anteriorly to the common response to all stimuli at 6 Hz and harmonics. Conventional sensor and source space analyses of evoked responses in the time domain further corroborated this result. Our results demonstrate that FPVS in combination with simultaneously recorded EEG and MEG may serve as an efficient localizer paradigm for human face categorization.

## Highlights

• FPVS allows high-SNR EEG/MEG recordings of face-selective brain responses.
• High comparable z-scores obtained for EEG and MEG in all but one participants.
• Face-selective responses right-lateralized in EEG.
• Face-selective responses bilateral but numerically right-lateralized in MEG.
• Strongest face-selective sources anterior to base frequency response.

## 1 Introduction

The speed and efficiency of our ability to categorize a large number of objects from visual input within a fraction of a second has been the focus of extensive neuroscientific research over the last decades (Cichy at al., 2014; Fabre-Thorpe, 2011; Lamme and Roelfsema, 2000; Palmeri and Gauthier, 2004; Riesenhuber and Poggio, 2002; Serre at al., 2007; Thorpe, 2009). Faces have received special interest because of their social relevance in humans, specific brain disorders, and distinctive brain signatures (Freiwald at al., 2016; Grill-Spector et al., 2017; Haxby et al., 1996; Rossion, 2014a; Sergent and Signoret, 1992). The neurotypical adult human brain quasi-automatically categorizes face stimuli at multiple levels: e.g., according to its emotional expression, sex, race, familiarity, etc. The most basic categorization level is that of the visual stimulus as a face, as opposed to its categorization as a non-face object. Although the categorization of a visual stimulus as a face may appear as a trivial task because it is achieved at astonishing speed and automaticity by a neurotypical human adult (Crouzet et al., 2010; Fletcher-Watson, Findlay et al., 2008; Hershler and Hochstein, 2005; Lewis and Edmonds, 2003), it is a challenging function for which artificial systems still lag well behind the human brain (e.g. Scheirer et al. 2014).

A fast periodic visual stimulation (FPVS) paradigm combined with electroencephalographic (EEG) recordings has been suggested as an efficient way to study multiple levels and types of face categorization (Rossion, 2014b), in particular the categorization of natural visual stimuli as faces (Rossion et al., 2015). FPVS is based on the principle of “frequency-tagging”, according to which stimuli presented at a periodic rate lead to periodic brain responses (Adrian and Matthews, 1934), which can be objectively identified and separated from noise in the EEG frequency spectrum (Regan, 1989; Norcia et al., 2015). The FPVS approach provides particularly high signal-to-noise ratio (SNR) responses, allowing short recording durations. In recent years, this paradigm has been increasingly used with numerous variable natural images of faces and nonface objects, providing face-selective responses that are generalizable across face exemplars and not accounted for by low-level visual cues contained in the image statistics (amplitude spectrum; (Rossion et al., 2015)). Moreover, previous studies presenting different exemplars of various object categories (e.g., houses and non-face body parts) have shown that their periodic category-selective responses are substantially smaller and different in topography and lateralization compared to faces (Jacques et al., 2016) (see also Hagen et al. 2020 for intracranial evidence).

Importantly, while this paradigm has been used extensively with EEG but also intracranial recordings (Jonas et al., 2016) and functional magnetic resonance imaging (fMRI) (Gao et al., 2018), it has not been used with magnetoencephalography (MEG). In addition, previous frequency-tagging MEG studies with face stimuli have not isolated face-selective responses (e.g. Baldauf and Desimone 2014) for generic responses to spatially overlapping faces or houses modulated by selective attention; Tsuruhara et al. (2014) to upright and inverted geometrical faces).

Surface EEG and MEG do not provide reliable information about the neural generators of the signals (e.g. Ahlfors et al. 2010). Intracranial recordings can provide information about the spatial localization and distribution of neural generators. However, a direct comparison of EEG and intracranial recordings is complicated by the latter's limited coverage of the brain and differences in their sensitivity to brain sources. While MEG, just as EEG, also suffers from the non-uniqueness of the so-called inverse problem (Sarvas, 1987) and therefore has only limited spatial resolution, the highest spatial resolution can be obtained using a combination of EEG and MEG recordings, as they are sensitive to different spatial aspects of the neural current distributions (Ahlfors et al., 2010; Goldenholz et al., 2009; Hauk et al., 2019; Henson et al., 2009; Molins et al., 2008).

Here, for the first time we apply the FPVS paradigm of generic face categorization with combined EEG/MEG recordings and source estimation. Our main aim is to (1) replicate the original EEG findings (Rossion et al., 2015) in MEG, (2) test whether MEG (gradiometers and magnetometers) is equally sensitive to face-selective FPVS responses as EEG, (3) and if so, use combined EEG and MEG responses to estimate their neural generators using distributed source estimation in individual head and source models. Of particular interest is the comparison between category-selective EEG and MEG signals in the frequency-domain in terms of their respective signal-to-noise ratios and relative values across individual brains (i.e., are face-selective MEG and EEG responses correlated across individual brains and if so to what extent?). Our source estimation results extend these findings and provide evidence for the neural generators of the face categorization response, especially with respect to their laterality.

## 2 Methods

### 2.1 Participants

Eighteen participants in the age range 19–35 years were recruited from the MRC CBU's volunteer panel database (9 identified themselves as female, mean age = 28.33). Two of them had to be excluded because we were not able to obtain their structural MRI images, and one was excluded because of large MEG artefacts during the recording session (likely due to a dental implant), leaving 15 datasets for the final analysis. All participants reported to be right-handed native English speakers, to have normal or corrected-to-normal vision, and to have no history of neurological or developmental disorders. They were monetarily reimbursed for their participation to the study. This study was approved by the Cambridge Psychology Research Ethics Committee.

### 2.2 Stimuli

**Fig. 1:**
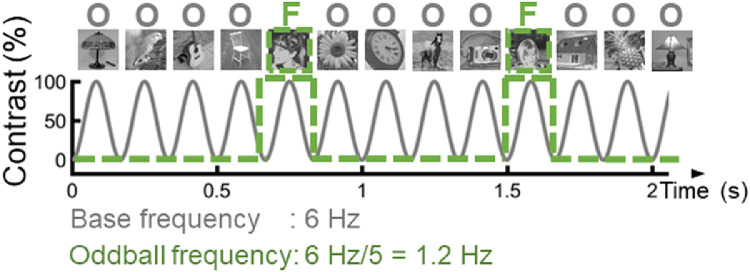
Illustration of the FPVS face-object paradigm. The same stimuli as in Rossion et al. (2015) were used. Natural images of objects were presented at 6 Hz following a sinusoidal contrast modulation. Unfamiliar face images were inserted every 5 stimuli, corresponding to a frequency of 1.2 Hz (= 6 Hz/5). Note that the face pictures displayed in the figure are different from those used in our experiment because of copyright reasons.

Our stimuli and experimental settings are close to those described in Rossion et al (2015). The stimulus set was the same as used in that study. It consisted of 200 black and white photographic images of various common objects (animals, plants, objects and houses) collected from the internet, and 50 photographs of unfamiliar faces. Importantly, all objects and faces were unsegmented, i.e., embedded in their original visual scene. The various objects and faces were centred, but they differed in terms of size, viewpoint, lighting conditions, and background (Fig. 1; Movie 1). The entire set of stimuli is available online at https://face-categorization-lab.webnode.com/resources/natural-face-stimuli/. The stimuli were converted to grayscale, resized to 200 × 200 pixels, and equalized in terms of pixel luminance and root-mean square contrast in Matlab. Importantly, given that this normalization is performed on the whole image, the faces in these natural images still purposely differed substantially in local luminance, contrast, and power spectrum. Shown on a screen at a distance of approximately 1.3 m in front of the participant, the stimuli subtended approximately 4° of visual angle.

### 2.3 Procedure

A schematic illustration of the FPVS paradigm as employed is presented in Fig. 1. Stimuli were presented to participants with Java. All stimuli were presented on a uniform grey background using sinusoidal contrast manipulation, from 0 to 100% to 0% for each stimulus (Rossion et al, 2015). Monitor refresh rate was 60 Hz.

Participants completed three runs of face-object stimulation. Each run consisted of pictures of objects presented at a base frequency of 6 Hz (166 ms per stimulus), and every fifth image was an unfamiliar face (frequency 1.2 Hz). A neural response that does not differ systematically between faces and non-face objects will project on the 6 Hz component of the EEG spectrum and its harmonics (12 Hz, etc.), whereas a differential response for faces (i.e., a face-selective response) will be reflected at 1.2 Hz and harmonics (2.4 Hz, etc.). Each run lasted for 64 s, including 2 s of fade-in and fade-out at the beginning and at the end of the sequence, respectively.

As in previous studies (e.g. Rossion et al. 2015), participants were instructed to perform a colour change detection task during the stimulus presentation period. Participants were asked to press a button with their right index finger when they perceived a change in the colour of the fixation cross which is presented in the centre of the screen. The colour change happened (randomly) 8 times per run and it lasted for 500 ms. The minimum difference in time between each colour change was 2 s. One participant's behavioural responses were only collected for one one-minute run due to a technical error.

This task was chosen because it is orthogonal to the experimental manipulation (i.e., face categorization), and has been used widely in previous FPVS studies (e.g., Liu-Shuang et al. 2014, Rossion et al. 2015 and see Rossion et al. 2020). Its purpose was to ensure that participants paid attention to the stimuli. This was confirmed, as performance was high with an average correct target detection rate of 95% (standard deviation 6%, min|max 83%|100%) and an average response time of 523 ms (SD 68 ms, min|max 412 ms|676 ms), which is comparable to previous studies (Retter et al., 2020; Rossion et al., 2015). The face-object runs were administered after 9 similar runs using word stimuli, the results of which will be reported elsewhere. We also ran two 2 min resting-state runs at the beginning and end of the recording session in order to compute noise covariance matrices for source estimation (see below).

### 2.4 Data acquisition

EEG/MEG data were acquired on an Elekta Neuromag Vectorview system (Elekta AB, Stockholm, Sweden), containing 306 MEG sensors (102 magnetometers and 204 gradiometers), and 70 EEG electrodes mounted on an Easycap cap (EasyCap GmbH, Herrsching, Germany). The EEG recording reference electrode was attached to the nose, and the ground electrode to the left cheek. The electrooculogram (EOG) was recorded from electrodes above and below the left eye (vertical EOG) and at the outer canthi (horizontal EOG). The sampling rate during data acquisition was 1000 Hz and an on-line band pass filter 0.03 to 330 Hz was applied. Prior to the EEG/MEG recording, the positions of 5 Head Position Indicator (HPI) coils were attached to the EEG cap (for head localisation inside the scanner and continuous movement tracking), 3 anatomical landmark points (two preauricular points and nasion) as well as the EEG electrodes and about 50–100 additional points that cover most of the scalp were digitised using a 3Space Isotrak II System (Polhemus, Colchester, Vermont, USA) for later co-registration with MRI data. Our data can be made available via the MRC Cognition and Brain Sciences Unit's data repository on request.

High-resolution structural T1-weighted MRI images were acquired in a 3T Siemens Tim Trio scanner at the MRC Cognition and Brain Sciences Unit (UK) with a 3-D magnetization prepared rapid gradient-echo sequence, field of view = 256 × 240 × 160 mm, matrix dimensions = 256 × 240 × 160, 1 mm isotropic resolution, repetition time = 2250 ms, inversion time = 900 ms, echo time = 2.99 ms, and flip angle = 9°.

### 2.5 Sensor space analysis

#### 2.5.1 Pre-processing

MEG data were subjected to spatio-temporal signal-space separation (SSS) implemented in the Maxfilter software (Version 2.2.12) of Elekta Neuromag to remove noise generated from sources distant to the sensor array (Taulu and Kajola, 2005; Taulu and Simola, 2006). The SSS procedure included movement compensation (locations recorded every 200 ms), bad MEG channel interpolation, and temporal SSS extension (with default buffer length 10 s and sub-space correlation limit 0.98). The origin in the head frame is chosen as (0,0,45) mm.

The following steps of analysis were performed in MNE-Python Version 0.20 software package (http://martinos.org/mne/stable/index.html) (Gramfort et al., 2013). After visual inspection of the raw data, bad EEG channels (determined by visual inspection) were interpolated using spherical harmonics and ‘accurate’ option in MNE-Python. On average we interpolated 1.8 EEG channels and 2.3 MEG channels (as above) per participant. A notch filter at 50 and 100 Hz was then applied, followed by a band-pass filter between 0.1 and 140 Hz. In order to remove eye movements and heart artefact an Independent Component Analysis (ICA) was computed, removing a maximum of 4 ICA components (2 for heart, 2 for eyes). The exact ICA procedure closely followed the examples provided for the MNE-Python software (https://martinos.org/mne/dev/auto_tutorials/plot_ica_from_raw.html), which uses the temporal correlation between ICA components and EOG channels as a criterion for the removal of ICA components.

#### 2.5.2 Frequency- and time-domain analyses

First, data of the three 60 s runs (with fade-in and –out periods removed) were averaged in the time domain to improve SNR, and a Fast Fourier Transform (FFT) with frequency resolution $0.08\bar{3}$ was applied. We divided the FFT spectrum into segments of +/- 0.75 Hz centred at the frequency of interest and its higher harmonics (e.g., 0.45–1.95 Hz for the 1.2 Hz peak; 1.65–3.15 Hz for the 2.4 Hz peak, etc.). Ten segments centred on the frequency of interest and the higher harmonics were then summed up (i.e., until 14.4 Hz) (Retter et al., 2021). For the face-selective frequency, i.e. 1.2 Hz, any multiples of the base frequency (i.e., 6 and 12 Hz) were excluded. In order to correct for the variations in baseline noise levels around each frequency of interest, the amplitude of neighbouring frequency-bins within a range of 0.75 Hz on each side was averaged and then subtracted from each frequency bin (Retter et al., 2020). A gap of one frequency bin on each side of the target frequency was included in case of remaining spectral leakage. The minimum and maximum values were also removed from the baseline interval. We applied the same summing and baseline correction procedure to the harmonics of the base frequency (6–120 Hz). Finally, Z-scores for face-selective and base stimulation frequencies were computed by dividing the baseline-corrected amplitudes by the standard deviation of the neighbouring bins.

Sensor space results are presented separately for the three sensor types employed in this study: EEG, gradiometers and magnetometers. The physics of EEG and MEG signal generation indicates that these sensor types do not have to produce the same pattern of results (e.g. with respect to laterality), and that their combination provides the most complete information for source estimation (Ahlfors et al., 2010; Hauk et al., 2019; Henson et al., 2009; Molins et al., 2008). The EEG measures the electric potential at the locations of individual electrodes in Volts. The MEG data in our study were measured by two sensor types of the MEGIN Vectorview system: magnetometers and planar gradiometers. Magnetometers measure the magnetic flux through a single coil in Tesla, while gradiometers measure the magnetic flux gradient (i.e. the difference between two adjacent coils) in two orthogonal directions in Tesla-Per-Meter. A magnetometer and two orthogonal planar gradiometers at the same location are sensitive to different source configuration (they have orthogonal “leadfields”). Thus, placing a combination of one magnetometer and two orthogonal planar gradiometers at each location of the sensor array results in optimal sampling of the magnetic field distribution with a given amount of sensors. These sensor types differ with respect to their sensitivity to electrical brain activity. While the sensitivity of all sensor types drops of with the distance between source and sensors, this drop-off is steeper for MEG than EEG and steeper for gradiometers than magnetometers (in a perfect homogeneous sphere a dipole at the sphere's centre does not produce any external magnetic field). MEG is relatively insensitive to radial dipolar sources (i.e. perpendicular to the scalp; in a perfect homogenous sphere these sources would not produce an external magnetic field), while EEG is more sensitive to radial compared to tangential dipolar sources. In simulations EEG has been shown to be more sensitive to spatially extended sources than MEG (Ahlfors et al., 2010). Thus, in this study we tested whether we can detect face-selective FPVS responses in each sensor type. Because this was the case (see below), we combined all sensor types for source estimation.

As there are two gradient measurements per sensor location, the values of each gradiometer pair will be plotted as the root-mean-square (RMS) per pair. Note that we will present all results as Z-scores, which are comparable across sensor types.

We will display results in the frequency domain for sensors with maximum Z-scores at the face-selective and base frequency, respectively. Because these responses are several times larger than our significance threshold (*t*=1.96), and for EEG they replicate previous findings, we do not consider the selection of peak sensors as “double dipping” (Kriegeskorte et al., 2009; Tiedt et al., 2016), but rather as the selection of sensors with the largest signals. In addition, we include the topographies of z-scores for face-selective and base frequencies as inlets, which are clearly non-random and consistent with activity arising from sources in posterior brain areas. For EEG, we ran a separate analysis with electrode groups close to those in Retter et al. (2020), which yielded qualitatively similar results. Responses will be considered to be significant when z-scores exceed 1.96 (*p*<0.05, 2-tailed).

In order to test for laterality differences in posterior EEG channels, we selected four electrodes that were close to those used in Retter et al. (2020) (i.e. P7/8, P9/10, PO7/8, P09/10 in the left/right hemisphere). No previous data exist from MEG experiments to reliably define sensor groups of interest. We therefore used generic groupings of 12 magnetometers and 24 gradiometers above left and right occipital brain areas^1^. We ran paired one-sided t-tests to assess statistical significance of differences between left and right sensor groups.

EEG and MEG data will then be combined for source estimation. Similarly, based on clear results in sensor space, we will interpret peak z-scores in posterior brain regions for our source estimates.

Evoked responses in the time domain were computed for face stimuli for a latency window from -200 ms before to 500 ms after stimulus onset, with a Notch filter at the base frequency and its harmonics (transition bandwidth 0.02 Hz).

### 2.6 Source space analysis

Source estimation was performed on combined EEG/MEG data using L2-minimum-norm estimation, as appropriate for data where the number and location of sources is not known a-priori (Hämäläinen and Ilmoniemi, 1984; Hauk, 2004). In the frequency domain, source estimates were computed for the summed topographies across harmonics for the face-selective and base frequencies, respectively. This was performed in MNE-Python software with standard parameters settings. We used individual MRI images for head-modelling. The MRI data were pre-processed in Freesurfer V6.0.0 (Fischl, 2012), and the head model (3-layer boundary element model) created in MNE-Python. We used L2-minimum-norm estimation without depth-weighting or noise-normalisation, and with a loose orientation constraint (ratio of variances between tangential and normal dipole components: 0.2). The data from different sensor types were combined for source estimating using a standard “whitening” approach implemented in the MNE-Python software. We used a regularisation parameter based on a SNR value of 3 (default in the software). The procedures for baseline-correction and z-scoring in the frequency domains as described above were also applied to source-space results. The same parameter settings were applied for source estimation of the evoked face-selective signals in the time domain.

The laterality of face-selective FPVS responses in source space was investigated for several regions-of-interest in posterior inferior temporal cortex from the HCP-MMP1.0 parcellation (Human Connectome Project Multi-Modal Parcellation version 1.0) (Glasser et al., 2016). While the “fusiform face complex” of this parcellation is of particular interest for our study, we also included surrounding regions due to the limited spatial resolution of EEG/MEG (see Fig. 6). For the same reason we do not consider a more fine-grained analysis of these parcels, some of which are in deeper areas of the inferior temporal lobe, meaningful for our purposes (Hauk et al., 2019; Krishnaswamy et al., 2017; Molins et al., 2008). We assessed statistical significance of brain activation between the two hemispheres using paired one-tailed t-tests.

## 3 Results

In the following, we will first present results in the frequency domain followed by evoked face-selective responses, and in each case we will first show sensor space results followed by source estimates.

**Fig. 2:**
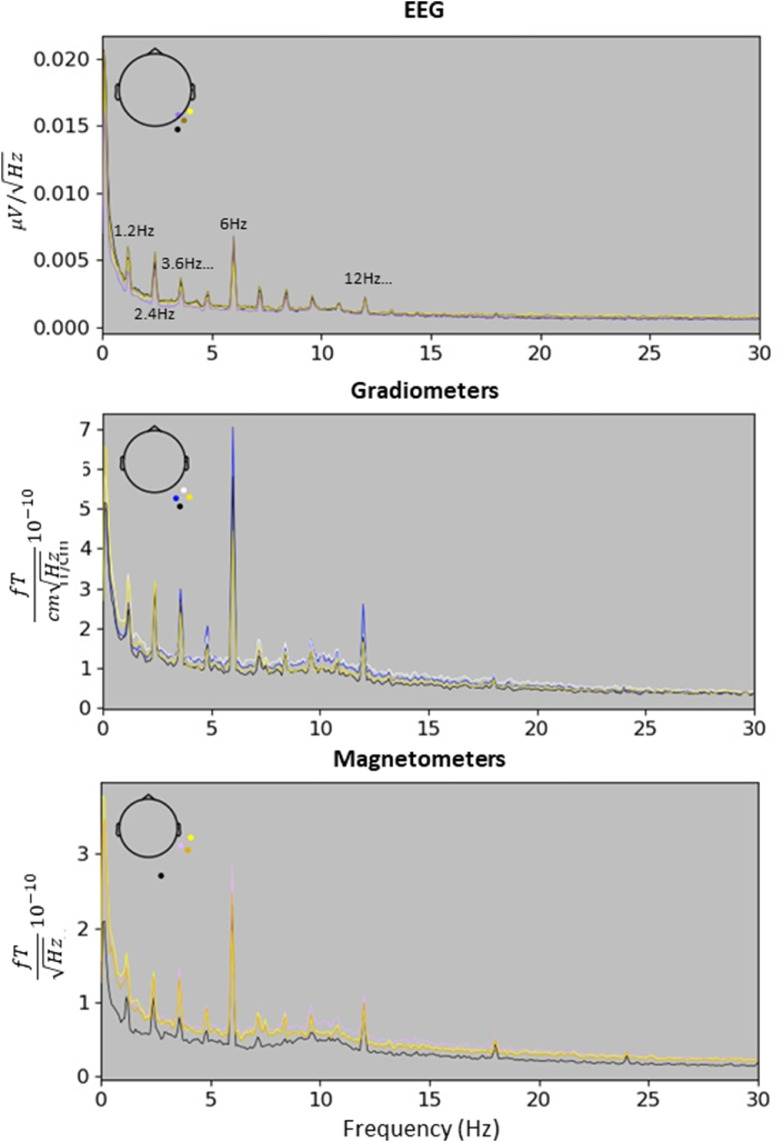
Power spectral densities of FPVS data for EEG, gradiometers and magnetometers. The peaks at multiples of 6 Hz correspond to the base frequency of visual stimulus presentation, and the peaks at multiples of 1.2 Hz correspond to face-selective responses. We present signals from four peak sensors determined at the face-selective frequency in the grand-average. Their positions are indicated in the inlet.

Fig. 2 shows the z-scored frequency spectra grand-averaged across all participants. We selected four peak sensors with maximum Z-scores (coloured lines) for each sensor types, i.e. EEG (top), gradiometers (middle) and magnetometers (bottom). The peaks at multiples of 6 Hz reflect the base frequency, or the common response to object and face presentation, and the multiples of 1.2 Hz the selective frequency of face presentation. While the peaks for the base frequency reflect brain responses driven by the periodic presentation of visual stimuli across object categories, the responses for the face frequency reflect discrimination between faces and objects, i.e. a categorization response (Rossion et al., 2015). All sensor types show face categorization responses in posterior sensors.

We analysed the lateralization of these responses separately for the three sensor types. For EEG, the t-test on posterior electrode groups revealed a right-lateralisation for face categorization responses (Left 3.24 vs Right 6.13, *t*(14)=-2.85, *p*(14)=0.006) as well as for base frequency responses (11.97 vs 20.48, *t*(14)= -2.43, *p*(14)=0.014). Gradiometers only showed a numerical but non-significant right-lateralization for face categorization (4.28 vs 5.14, *p*=0.16), but this was significant for the base frequency (21.53 vs 26.77, *p*<0.0002). A similar non-significant lateralization pattern was found for magnetometers for face categorization (3.79 vs 4.18, *p*=0.26), which was marginally significant for the base frequency (18.71 vs 22.69, *p*=0.06). Thus, while we replicated previous right-lateralization of face categorization responses for EEG, these responses appeared numerically but non-significantly right-lateralized in MEG. Note that for the unspecific base frequency response a right-lateralization was also reported in previous studies for EEG (e.g. Rossion et al. (2015)).

**Fig. 3:**
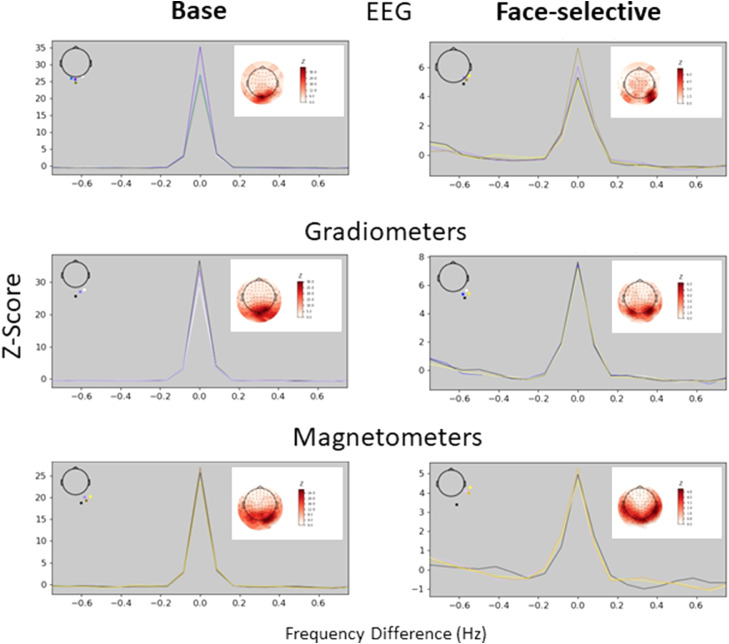
Sum of Z-scored frequency spectra epoched around harmonics of base (left) and face-selective (right) frequencies. Data are shown for four peak sensors (coloured lines) within EEG (top), gradiometers (middle) and magnetometers (bottom). Peak sensors were determined from the grand-mean across all participants, and their locations are shown in the top-left inlets. The top-right inlets present sensor space topographies for face-selective and base frequency responses summed across harmonics. The peak at 0 Hz for face-selective responses indicates reliable discrimination between faces and objects. All sensor types show reliable face discrimination responses.

Fig. 3 presents the Z-scored summed frequency spectra epoched around harmonics of base (top) and face-selective oddball (bottom) frequencies. As before, results are shown for peak sensors of each sensor type separately. The peak centred at 0 Hz for oddball responses confirms the presence of discrimination responses between faces and objects in all sensor types.

**Fig. 4:**
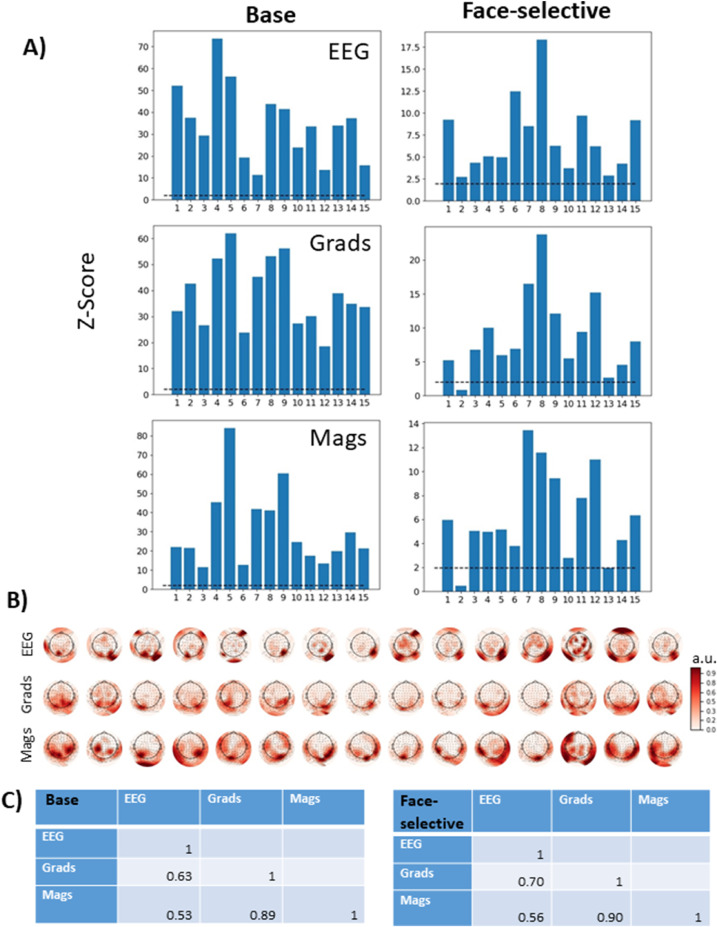
Z-scores for individual participants. (A) Root-mean-squared Z-scores for peak channels of different sensor types for base and face-selective responses. The horizontal lines represent a z-score of 1.96. (B) Individual topographies of face-selective responses summed across harmonics and scaled to their individual maxima. (C) Correlations of peak z-scores for sensor types across participants. All sensor types show reliable face discrimination responses for most participants.

Fig. 4 presents Z-scores for individual participants. Fig. 4A shows Z-score values (root-mean-squares of peak channels) for base and face-selective responses for all individual participants. Fig. 4B presents the corresponding topographies, scaled to their individual maxima. For EEG, all 15 participants show significant face categorization responses, and for MEG sensors there is only one participant who does not. However, the MEG topographies for this participant (S2) in Fig. 4B do show bilateral maxima in posterior areas, consistent with signals originating in visual brain areas. It is possible that for this particular participant the orientation of the current sources was approximately radial with respect to the skull, thus allowing detection in EEG but staying below significance level in MEG. In sum, this demonstrates that all sensor types show face categorization responses for most participants.

Fig. 4C shows the correlations of Z-scores of Fig. 4A among sensor types across participants. Confidence intervals were estimated based on standard deviations of Fisher's Z-transformed correlations^2^. For MEG sensors these correlations were 0.9 (face-selective; low 0.72, high 0.97) and 0.89 (base; 0.7, 0.96). For EEG and gradiometers the correlations were 0.7 (0.3, 0.89) and 0.63 (0.17, 0.86), and for EEG and magnetometers 0.56 (0.07, 0.83) and 0.53 (0.03, 0.82). While confidence intervals partially overlap, this provides evidence that EEG and MEG carry independent information. That is, a large face-selective response in EEG is not necessarily associated with a large face-selective response in the concurrent MEG recording. This is a major justification for their combination in source estimation.

In order to test whether independence between EEG and MEG also holds with respect to laterality, we also correlated the laterality values (i.e. left-right differences for the sensor groups used in the laterality analysis above). These correlations between EEG and MEG were indeed close to 0 (EEG vs Gradiometers: 0.03, 95% confidence interval (-0.49, 0.53); EEG vs Magnetometer: 0.06 (-0.47, 0.56)), and clearly lower than between the MEG sensor types (Gradiometers vs Magnetometers: 0.74 (0.37, 0.91)). This confirms that EEG and MEG carry independent information about the lateralization of face-selective FPVS responses.

**Fig. 5:**
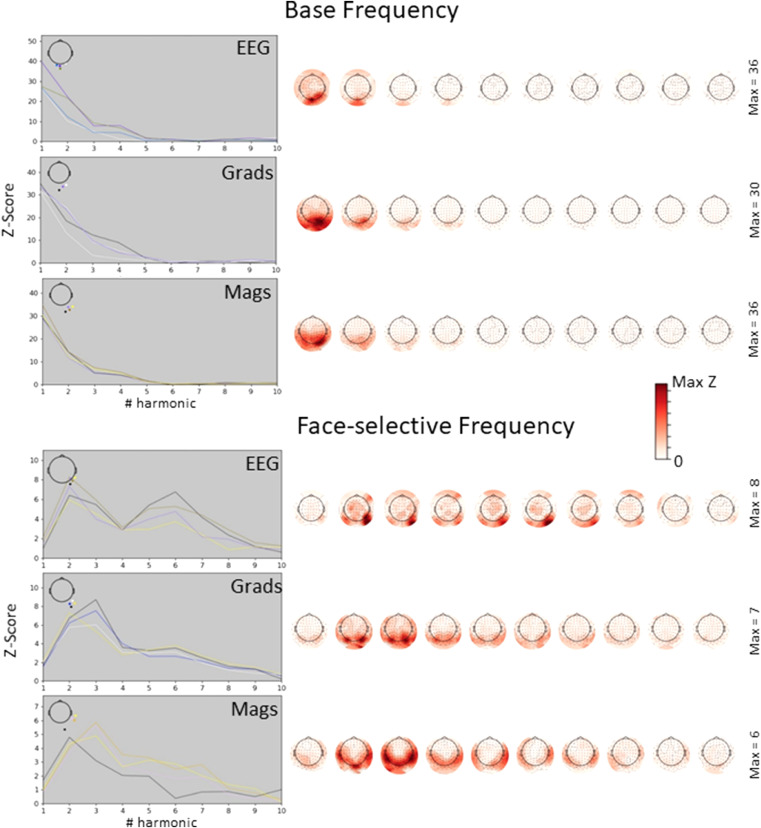
Z-score amplitudes and topographies across harmonics for different sensor types. Data are shown for base (top) and face-selective (bottom) frequency. The left panels present amplitudes for four peak sensors (coloured lines) within EEG (top), gradiometers (middle) and magnetometers (bottom). On the right we show the corresponding topographies (colour scheme indicated in the middle, with maximum Z-scores to the right of the topographies). Amplitudes decrease across the first 10 harmonics for all sensor types.

Fig. 5 displays the peak Z-scores and topographies for individual face-selective and base frequency harmonics in more detail. For the base frequency, z-scores decrease rapidly across harmonics: The second harmonic's Z-score is already less than half that of the fundamental or first harmonic. For the face-selective frequency, z-scores increase for the second harmonic (2.4 Hz) compared to the fundamental frequency. This is likely due to high noise level around the fundamental frequency of 1.2 Hz.

**Fig. 6:**
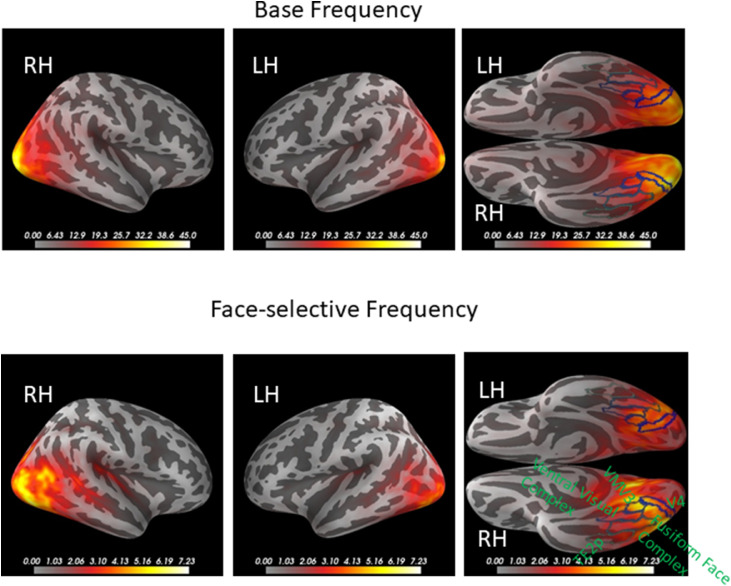
Z-scored L2-MNE source estimates for summed amplitudes across harmonics for base (top) and face-selective (bottom) frequency. Distributions are shown on inflated cortical surfaces in lateral view (first two columns, right and left hemisphere, respectively) and ventral views (3rd column). The regions-of-interest (green outlines) were chosen from the HCP-MMP0.1 parcellation (VMV3: Ventro-medial visual area 3, TE2p: Temporal area 2 posterior). The peaks in both hemispheres are more anterior for face-selective compared to base frequency responses. RH: right hemisphere; LH: left hemisphere. (For interpretation of the references to color in this figure legend, the reader is referred to the web version of this article.)

The summed and Z-scored L2-minimum-norm source estimates obtained from combined EEG and MEG data for activity at the base and face-selective frequencies are displayed in Fig. 6. Peak activity is confined to posterior brain regions in both cerebral hemispheres, but is more widespread in the right compared to the left hemisphere. The most anterior peaks for z-scores at the base frequency occurred in visual brain area V4 (based on HCP-MMP1.0 parcellation (Glasser et al., 2016)), while for face categorization responses peaks were located more anteriorly, overlapping with the Fusiform Face Complex (FFC) and Ventral Medial Visual area 3 (VMV3). Because of localization biases inherent in EEG/MEG data, we also tested for laterality effects in the neighboring areas depicted in Fig. 6 using paired one-tailed t-tests. For the base frequency we found significant right-lateralization in V4 (Left 45.8 vs Right 61.3, *t*=-2.29, *p*<0.05) and FFC (33.8 vs 26.5, *t*=-1.86, *p*<0.05), but not in other areas (all *p*>0.2). Laterality did not approach significance for face-selective responses in either FFC (4.5 vs 5.3, *t*=-0.86, *p*=0.2), VMV3 (3.3 vs 3.6, *t*=-0.48, *p*=0.32) or surrounding areas (all *p*>0.2). Thus, while our source estimates revealed significant right-lateralization for the base frequency as in the sensor space data for all sensor types, face categorization responses were more bilateral as in the MEG sensor data.

**Fig. 7:**
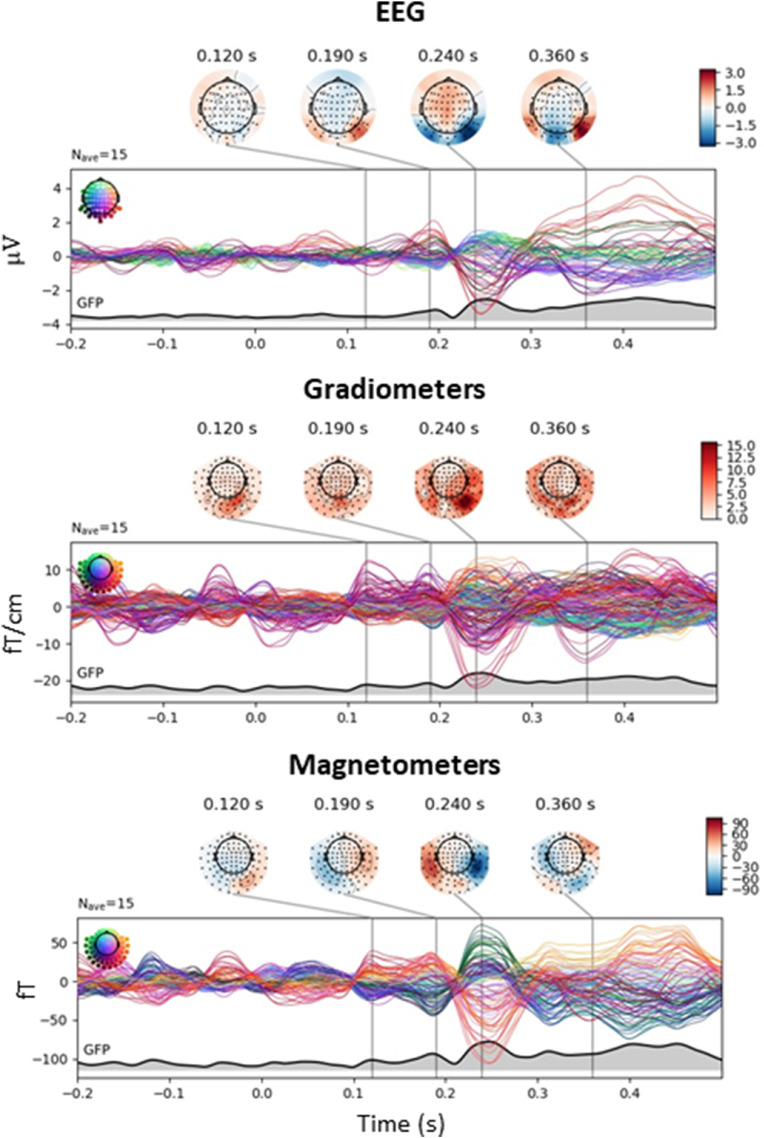
Time domain responses to face onsets in the fast periodic sequence. Note that the 0.0 s time point indicates the onset of the sinusoidal contrast modulation, with 100% contrast reached at 83.3 ms at 6 Hz (fifth frame at 60 Hz). Evoked responses are shown with notch filtering at the base frequency and its harmonics (i.e., isolating face-selective responses) and for different sensor types. Topographies are shown for selected latencies (indicated by black lines). All sensor types show reliable evoked responses in posterior sensors. GFP: Global Field Power.

We also analysed our data using a conventional time-domain approach. The evoked responses based on time-domain averaged for face stimuli are shown in Fig. 7. These responses reflect face-selective brain activity in the time domain. Scalp topographies are included at four peak latencies (chosen based on previous EEG reports, Rossion et al. (2015) as well as by visual inspection of the time courses in order to use one latency per peak across sensor types). For the gradiometer topographies we computed the root-mean-square (RMS) value for the two gradiometers at every sensor location. Considering the delay in stimulus onset due to the sinewave contrast modulation used (see Retter et al. 2018), all three sensor types exhibit evoked responses with peaks around 120, 190, 240 ms and a broader peak around 400 ms (see also Rossion et al. 2015). The topographies associated with these peaks are consistent with brain activity in posterior brain areas.

**Fig. 8:**
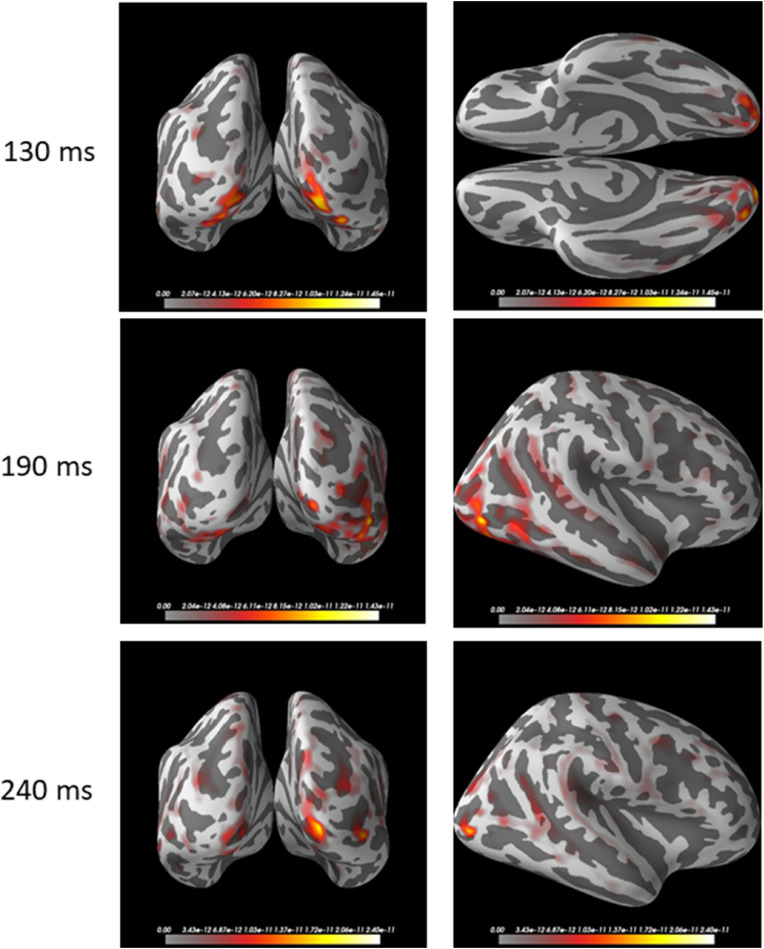
Grand-averaged L2-MNE source estimates for face-selective responses in the time domain. Source distributions are shown on an inflated cortical surface in caudal (left column) and ventral or right lateral (right column) view, for three different latencies (rows).

This is confirmed in the L2-minimum-norm estimates in Fig. 8. Brain activity in posterior brain is stronger and more widespread in the right hemisphere. Interestingly, while face-selective brain activity at 190 ms is more anterior to activity at 130 ms, it appears to move back to the occipital pole at 240 ms.

## 4 Discussion

We used the fast periodic visual stimulation (FPVS) paradigm with combined EEG and MEG to study the neural sources and dynamics of automatic face categorization processes in the human brain. We replicated previous results obtained with EEG (Or et al., 2019; Retter et al., 2020; Rossion et al., 2015), and show that MEG produces similar signal-to-noise ratios for face-selective FPVS responses as EEG. For the first time we presented distributed source estimates based on combined EEG and MEG recordings in a FPVS paradigm. The sources for general visual and face-selective brain responses were plausibly localized into posterior visual brain areas. The dominant sources were more anterior and at least numerically right-lateralized for face-selective brain responses, in line with extensive neuroimaging evidence (e.g. Grill-Spector et al. 2017, Jonas et al. 2016, Kanwisher et al. 1997, Rossion et al. 2012 and Sergent and Signoret 1992) and most importantly with intracerebral recordings obtained with the exact same stimulation paradigm (Hagen et al., 2020; Jonas et al., 2016). The sources for evoked responses in the time domain showed recurrent activation patterns within the first 250 ms after stimulus onset. All of our individual participants except one in MEG showed significant face-selective responses. We obtained our results based on data acquired in a very short amount of time (about three minutes). Thus, the FPVS paradigm can be used as an efficient localizer for face categorization brain activity in both EEG and MEG.

Since their sensors are sensitive to different source distributions (i.e., their leadfields are linearly independent), EEG and MEG signals provide complementary information. Importantly, the FPVS method provides an objective definition (i.e., at the experimentally-defined frequency) and quantification (i.e., sum of significant harmonics) of the response in the frequency domain, allowing a fair comparison of these signals across modalities. Interestingly, Z-scores were high and comparable across modalities (Figs. 2 and 3). While the patterns of harmonics differed slightly between MEG and EEG, these patterns reflect the shape of the response in the time domain (Retter and Rossion, 2016; Retter et al., 2021), which is indeed different across MEG and EEG signals (Fig. 7), albeit being dominated by a large negative component peaking at around 250 ms (with a delay in stimulus onset due to the sinusoidal contrast stimulation mode). In our study the correlation of amplitudes for face-selective brain responses between EEG and MEG sensors across participants was high but not perfect, and significantly lower than unimodality (MEG-MEG) correlations. This indicates that EEG and MEG signals reflect partially different source configurations, e.g., varying in source locations and spatial extent, or in other words the same sources project differently to the scalp to be recorded in MEG *vs.* EEG (Ahlfors et al., 2010; Ahlfors et al., 2010). Either way, this means that combined EEG and MEG provide a more complete picture of face categorization responses recorded outside the brain and higher spatial resolution than each of these measurement modalities on their own (Hauk et al., 2019; Molins et al., 2008).

We observed right hemispheric (RH) lateralization of the face-selective response for EEG, in line with results from previous studies (Or et al., 2019; Retter et al., 2020; Rossion et al., 2015) and with the right hemispheric lateralization of the face-selective N170 evoked by the sudden onset of a face (see Bentin et al. 1996, Rossion and Jacques 2011 for review). This right-lateralization was only numerically present in both MEG sensor types, which showed more bilateral face categorization responses. Importantly, the correlation of laterality values between EEG and MEG was low, providing further evidence that EEG and MEG carry different information about face-selective FPVS responses. Thus, we combined EEG and MEG for source estimation in order to analyse laterality effects in source space. The right lateralization in our EEG data and the bilateral MEG responses may reflect the fact that the overall neural sources are more radial in the right compared to the left hemisphere, but their tangential component is more bilateral or even left-lateralized. This could be due to the fact that a substantial amount of face-selective activity, in particular in this paradigm, may occur in the medial bank of the OTS, as shown with human intracerebral recordings (Hagen et al., 2020; Jonas et al., 2016), contributing to radial dipoles pointing to occipito-temporal EEG electrodes on the scalp (see Fig. 8 in Bentin et al. 1996). Moreover, right lateralized face-selective activity in the Mid-Fusiform Sulcus (Weiner, 2019), in the inferior occipital gyrus (see the simultaneous intracerebral-scalp recording study of Jacques et al. (2019)) as well in the anterior occipito-temporal sulcus (OTS) and anterior collateral sulcus (COS) (regions that are usually “invisible” in fMRI due to large magnetic susceptibility artefacts but show large face-selective responses in electrophysiology (Hagen et al., 2020; Jonas et al., 2016)) may also generate radial dipoles that would all contribute to the stronger right lateralization found in EEG than in MEG in the present study. This combination of contributions from these predominantly radial and tangential (e.g., lateral fusiform gyrus) sources further justifies the combination of EEG and MEG for source estimation.

Our source estimates also only showed a numerical but non-significant right-lateralization of face categorization responses. To our knowledge, MEG studies of face processing have only rarely reported a RH advantage in absolute responses to face stimuli (e.g. Lee et al. 2005 and Tiedt et al. 2013 in male subjects only) let alone for *face-selective* responses (i.e., no RH advantage in e.g., Deffke et al. 2007, Furey et al. 2006, Halgren et al. 2000, Linkenkaer-Hansen et al. 1998 and Liu et al. 2010, although see the recent study of Fan et al. 2020). Our source estimation results also produced more bilateral, but numerically right-lateralized, face categorization responses. Interestingly, a previous study comparing brain activation to faces and scrambled faces found right-lateralized difference activation for source estimates based on EEG, but more bilateral activation for the combination of EEG and MEG (Henson et al., 2009). It is noteworthy that our source estimates for the base frequency did reveal right-lateralized brain responses, in line with the sensor space data in all sensor types. Thus, our analysis was sensitive to lateralization effects in source space.

The FPVS paradigm provides an efficient tool to study the neural sources of face-selective responses in more detail in the future. Due to the short acquisition time and low task demands, it can be an efficient localizer for face-selective responses especially in clinical participant populations. Our source estimation results also provide the basis for future analyses on brain connectivity (Anzellotti and Coutanche, 2018; Basti et al., 2020; Palva and Palva, 2012) or brain-area-specific decoding (Kietzmann et al., 2019; King and Dehaene, 2014; Kriegeskorte, 2011; Stokes et al., 2015) of different stimulus types.

## CRediT authorship contribution statement

**O. Hauk:** Conceptualization, Methodology, Software, Formal analysis, Investigation, Visualization, Writing – original draft, Writing – review & editing, Supervision, Project administration. **G.E. Rice:** Conceptualization, Investigation, Resources, Supervision, Project administration. **A. Volfart:** Conceptualization, Software, Investigation, Resources, Writing – review & editing. **F. Magnabosco:** Investigation, Resources, Writing – review & editing. **M.A. Lambon Ralph:** Conceptualization, Writing – review & editing, Funding acquisition. **B. Rossion:** Conceptualization, Resources, Writing – review & editing, Funding acquisition.

## Footnotes

1 ‘Left-occipital’ and ‘Right-occipital’ in MNE-Python's function mne.read_selection().

2 Corresponding Python code: https://zhiyzuo.github.io/Pearson-Correlation-CI-in-Python/.
